# One Health: An inclusive framework to curb the COVID-19 pandemic

**DOI:** 10.17179/excli2021-3615

**Published:** 2021-03-26

**Authors:** Zeeshan Ahmad Bhutta, Muhammad Fakhar-e-Alam Kulyar, Ayesha Kanwal, Ashar Mahfooz, Moazam Ali, Kun Li

**Affiliations:** 1 The Royal (Dick) School of Veterinary Studies, University of Edinburgh, Easter Bush Campus, Midlothian, EH25 9RG, Scotland, United Kingdom; 2 College of Veterinary Medicine, Huazhong Agricultural University, Wuhan, 430070, PR China; 3 Institute of Biochemistry, Biotechnology and Bioinformatics, The Islamia University of Bahawalpur, Pakistan; 4 Department of Clinical Medicine and Surgery, University of Agriculture, Faisalabad, Pakistan; 5 Institute of Traditional Chinese Veterinary Medicine, College of Veterinary Medicine, Nanjing Agricultural University, Nanjing 210095, China

## ⁯⁯

***Dear Editor,***

SARS-CoV-2 virus is the responsible etiological agent of COVID-19 disease that was started from the Wuhan city of China, ultimately resulting in a global pandemic (WHO, 2020[9]). Genome sequencing analysis revealed that the bats are responsible for mutation, resulting in SARS-CoV-2. These bats are responsible for direct (bats to humans) and indirect transmission (unknown intermediate host) of virus to humans (Guo et al., 2020[3]; Oude Munnink et al., 2020[7]). However, this is not the first time that an unidentified virus emerged due to close contact with humans and wild animals. For example, the Severe Acute Respiratory Syndrome (SARS) pandemic in 2002-2003 was tracked down to interaction of civets with humans that were infected from bats (Kan et al., 2005[5]). It is estimated that 75 % of infectious diseases have always been originating from animals (Gebreyes et al., 2014[2]).

Humans are creating the environment, suitable for such threats to spread and sustain. Anthropogenic modifications, comprising the climate change, population growth, globalization, habitat destruction and interaction with wildlife (for food or trade) are the substantial drivers for the spread of infectious and/or zoonotic disease, ultimately leading to a serious threat to the economic development of the nation (Whitmee et al., 2015[8]). For example, the use of forest land for the agriculture use brings animals closer to humans. 

According to the WHO, One Health is “an approach to design and implement programs, policies, legislation and research in which multiple sectors communicate and work together to achieve better public health outcomes” (WHO, 2017[10]). One Health acknowledges that the health and welfare of humans, animals and environment are interlinked. It involves a collaborative effort of experts from a broad range of sectors including animal health, human health, plant health and environment by sharing information and coordination among such sectors.

**Figure 1:**
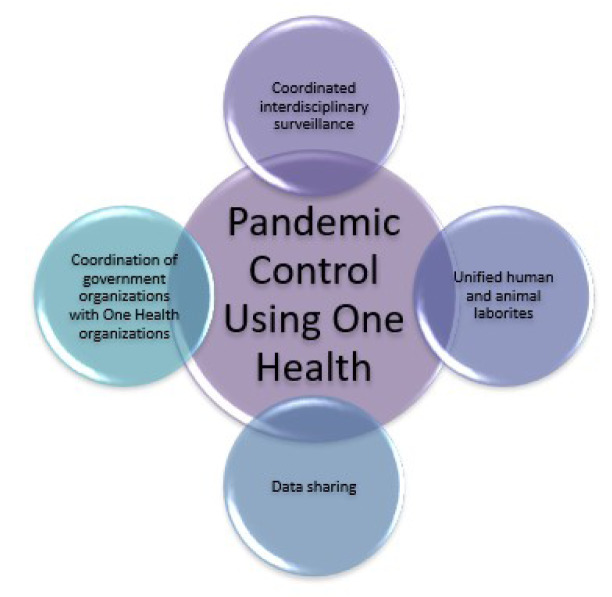
Schematic illustration of the possible solutions to combat the problem of current and future pandemics using One Health approach

One Health can be the supreme framework to tackle the COVID-19 because it has a zoonotic origin. One Health framework tries to lower the risk and alleviate effects of health crisis. Following steps can be adopted by the international community to combat the problem of current and future pandemics:

1. A coordinated interdisciplinary surveillance program throughout the pandemic for the sake of studying the transmission intensity, disease spread patterns, and geographical spread.
2. Set up unified human and animal laboratories in order to conduct the integrated studies in the pathogens to know their pathogenicity and spread pattern.
3. The data sharing regarding ideas, guidelines, protocols, diagnosis, results interpretation, and reporting system of COVID-19 and other zoonotic diseases. It will result to maintain the standards being adopted worldwide for disease diagnosis and surveillance.
4. Coordination of government organizations with One Health organizations to speed up disease prevention (Figure 1(Fig. 1)) (Mushi, 2020[6]).

Due to financial constraint, One Health approach will be advantageous in fight against the COVID-19 because it permits costs apportionment in cross disciplinary within accountable ministries. The operating costs for separate human and animal laboratories are higher than if the two areas work together and share resources (Häsler et al., 2013[4]). One Health approach will result into control spread of disease and decrease the economic as well as social burden caused by the COVID-19 pandemic through the collaborative effort of multiple sectors (Bonilla-Aldana et al., 2020[1]). The knowledge acquired from employing the One Health during pandemic will be helpful in threat reduction and control of emerging and re-emerging infectious diseases.

Current, COVID-19 pandemic is a golden chance for the world to adopt and emphasize the One Health approach and relocate resources to monitor and surveillance of disease. Governments should engage the different communities to spread awareness about the pandemic and One Health approach, because without the involvement of local communities, it would be impossible to tackle the problem.

Conclusively, despite the fact that COVID-19 has already been spread in numerous areas of the world, enforcement of One Health approaches would be a possible approach to control the spread of COVID-19. Now more than ever, there is a need to adopt, invest in and embrace the One Health approach, as it can help with the current COVID-19 pandemic as well as mitigate the threat of other infectious diseases.

## Notes

Zeeshan Ahmad Bhutta, Muhammad Fakhar-e-Alam Kulyar and Ayesha Kanwal contributed equally as first authors.

Zeeshan Ahmad Bhutta and Kun Li (Institute of Traditional Chinese Veterinary Medicine, College of Veterinary Medicine, Nanjing Agricultural University, Nanjing 210095, China; E-mail: lik2014@sina.com) contributed equally as corresponding authors.

## Conflict of interest

None.

## Authors’ contribution

All authors developed the idea and contributed to the final version of the manuscript equally.
